# Wettability of zinc oxide nanorod surfaces

**DOI:** 10.1039/c9ra05378f

**Published:** 2019-11-22

**Authors:** Hajar Ghannam, Adil Chahboun, Mireille Turmine

**Affiliations:** Sorbonne Université, CNRS, Laboratoire Interfaces et Systèmes Electrochimiques (LISE) 4, place Jussieu 75005 Paris France ghannam11hajar@gmail.com; Université Abdelmalek Essaadi, FST Tanger, Laboratoire Couches Minces et Nanomatériaux (CMN) 90000 Tanger Morocco

## Abstract

In this work, we have studied the wettability of zinc oxide (ZnO) nanorods grown on fluorine-doped tin oxide (FTO) by highlighting the effect of polar and non-polar ZnO facets on contact angle (CA) results. The variation in the wettability behaviors of the synthesized surfaces is mainly related to physical and chemical surface texturing which influenced the liquid drop penetration. Indeed, three main penetration states can be deduced: total, partial, and null-penetration. Where, low CA (100.9°) with high contact angle hysteresis (CAH) (13°) is observed for total penetration of the liquid drop. While, high CA (139.6°) with low CAH (7°) is observed for null-penetration. Moreover, we have found that the chemical texturing of ZnO, especially the hydrophilicity of ZnO tips, responsible for liquid drop sticking, prevents the liquid slipping over the surface. In order to promote the liquid rolling on the ZnO surface, we reported the physical modification of the ZnO structures. Therefore, the rolling of the liquid drop on the inclined surface of ZnO is achieved by using a new structure based on double scale roughness. This surface exhibits superhydrophobic behavior with a CA of 153° and CAH of 3°.

## Abstract 2

We have studied the wettability of zinc oxide (ZnO) nanorods grown on fluorine-doped tin oxide (FTO) by highlighting the effect of polar and non-polar ZnO facets on contact angle (CA) results.

## Graphical abstract

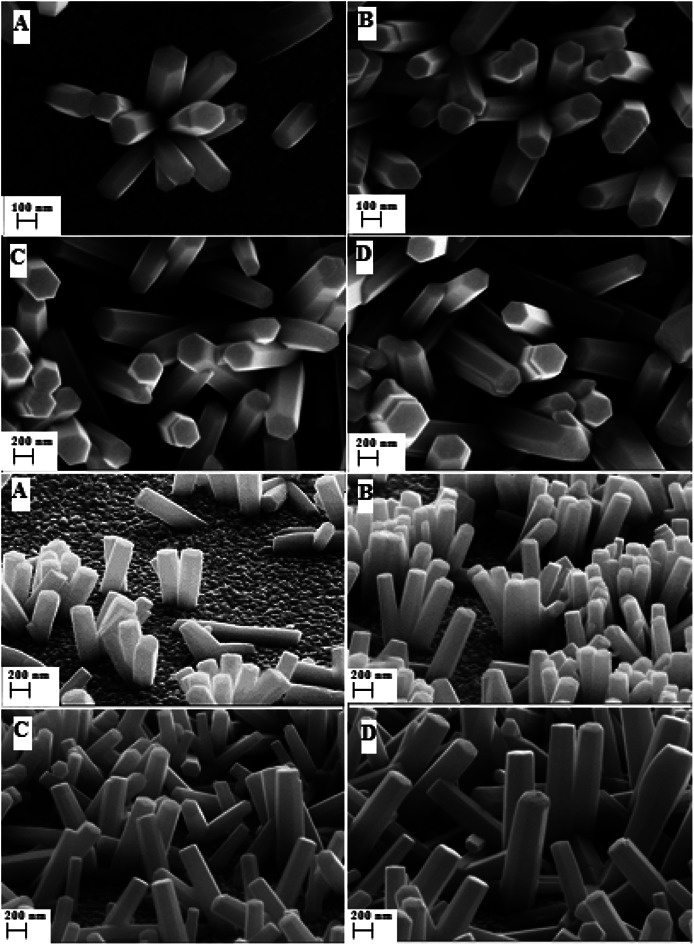

## 1 Introduction

The wettability is a surface property which describes the interaction between a liquid and a solid surface. It is quantified and qualified through the contact angle (CA) formed by a liquid droplet put onto a solid surface. Indeed, the CA value mainly depends on the surface tension of the solid substrate and its roughness.^1,2^ For a CA higher than 150°, no wetting state, the liquid beads upon the surface. In this case, the surface is named super-hydrophobic. The extreme water properties shown on super-hydrophobic surfaces such as lotus leaves or animal skins are due to high surface roughness and low surface energy coating.^3–5^ Conversely, a super-hydrophilic surface allows a liquid drop to spread out, wetting state, forming CAs approaching 0°. Super-hydrophilic surfaces are characterized by high surface energy coating.

Three main models are used to predict the liquid drop behavior. Young’s model is applied for an ideal surface, *i.e.*, a smooth and flat chemically homogeneous surface.^1^ Young’s contact angle is a result of the thermodynamic equilibrium of the free energy at the solid–liquid–vapor interface. Wenzel modified Young’s equation and proposed a model describing the contact angle at a rough surface by considering that the liquid completely penetrates into the rough surface grooves.^2^ According to this model, the roughness of the surface plays an amplifier role in the wettability behavior. Cassie and Baxter (CB) developed a model for a composite surface, made of a substrate and air like a woven polymer or a porous material.^6^ The CA depends on the ratio of the element surface composition which can be calculated using the CB model.

The wettability of zinc oxide (ZnO) is quite particular ascribed to its several properties. On one hand, the wurtzite ZnO structure is non-centrosymmetric. This contributes to a different facets polarity. The facets of (0001) and (0001̄) planes are considered as polar surfaces.^7^ Therefore, the polar facets are characterized by high surface energy which leads to the spreading out of the liquid. However, the facets of (11̄00) and (112̄0) planes are non-polar surfaces. The non-polar facets have a low surface energy favoring the liquid drop retraction and a thermal treatment makes them more hydrophobic but the hydroxyl groups of non-polar facets termination lead to hydrophilic behavior in the initial state.^8,9^ Thanks to the preferential growth of ZnO along the *c* axis, the non-polar facets are more dominant. Therefore ZnO exhibits a hydrophobic behavior.^10^ On the other hand, ZnO has a large band gap (3.3 eV), and therefore under UV light, photon adsorption generates pairs of electrons–holes. At the surface, the pairs of electrons–holes react with the liquid leading to its spreading. Moreover, the phenomenon of bi-wetting of ZnO nanorods with UV light/dark is reversible.^10,11^ Also, it is important to note that radicals produced by photo-catalytic reactions are strong agents for degrading organic pollutants. The wettability behavior and photocatalysis property of ZnO serves to its application as a self-cleaning coating.^12,13^

The structural property of ZnO thin films has a major influence on wettability behavior as on electrical, optical, and photocatalytic properties.^14–19^ Several works have modified the surface of ZnO physically and/or chemically in order to reach super-hydrophobicity. Indeed, organic molecules are the most nominated candidates in the manufacture of self-cleaning surfaces based on super-hydrophobic behavior. These molecules with low surface energy largely increase the contact angle value, which can sometimes reach values higher than 170°.^20^ Furthermore, the use of silicic molecules such as octadecylsilane have been very successful in the manufacture of super-hydrophobic surfaces, but they remain expensive and toxic.^21^ However, fatty acids are cheaper, non-toxic and more prevalent in nature. They have proven to be more effective. Badre *et al.*^35^ found that the modification of ZnO nanorods with stearic acid leads to a CA of 176°, whereas for elaidic and oleic acid a decrease in the contact angle was observed up to a value of 140°. But the chemical modification of ZnO surfaces with organic molecules leads to losing its photocatalytic property which serves to degrade organic pollutants. However, through physical modification the ZnO surfaces can reach super-hydrophobicity and retain its photocatalytic property. Zhang *et al.*^24^ showed by synthesizing ZnO with a controllable structure that the CA of a micronanobinary structure composed of microscale spheres and nanoscale protrusion binary structures of ZnO (170°) is greater than that of the nanostructure of ZnO protrusion structures (162°) and microscale of ZnO sphere structures (156°). Kim *et al.*^9^ have synthesized a high ordered ZnO nanorod surface which induces a CA of 154°. Despite the super-hydrophobicity of this surface, the liquid drop adheres to the surface.

In this paper, we present a detailed work on understanding the wettability of ZnO nanorod surfaces. ZnO nanorods with different sizes were synthesized using an electrochemical method.^25^ The fraction of the facets constitutive surface elements and the roughness state of the surfaces were estimated through the morphology of the electrosynthesized surfaces analyzed by field emission scanning electron microscopy. Moreover, the surface wettability is quantified by measuring the CAs. The CAs results were discussed as a function of chemical and physical surface properties. The variation of surface properties was attributed to liquid drop transition from the Wenzel state to the Cassie–Baxter state. This transition was accompanied by a significant variation in the contact angle hysteresis (CAH) ascribed to pinning and sticking of the liquid drop on the surface. However, the rolling of the liquid drop is achieved by modifying the roughness of the surface.

## 2 Experimental section

For ZnO electrodeposition we used an experimental setup consisting of a classical cell of three electrodes. A zinc rod was used as a counter electrode (CE), a saturated calomel electrode acts as a reference electrode (RE) and FTO glass (from Solems, 80 nm thickness) served as a working electrode (WE). The WE was cleaned before use by acetone and ethanol respectively for 10 min each, in an ultrasonic bath, afterwards it was rinsed with distilled water. These three electrodes were connected to a Potentiostat/Galvanostat (Gamry 600+) and vertically immersed in an aqueous electrolyte containing the ionic species necessary for ZnO formation (ZnCl_2_: MERCK, purity > 95%, KCl: VWR International, purity 99.0100.5%, Al(NO_3_)_3_: Sigma-Aldrich, purity > 99,99%, and dioxygen). The aqueous electrolyte was stirred constantly using a magnetic stirrer and its temperature was kept constant over the electrodeposition time by using a cryostat bath. To electrodeposit ZnO, we used potentio-static conditions where the ZnO reduction was implemented at a constant potential of −1 V/SCE according to the following reaction:1
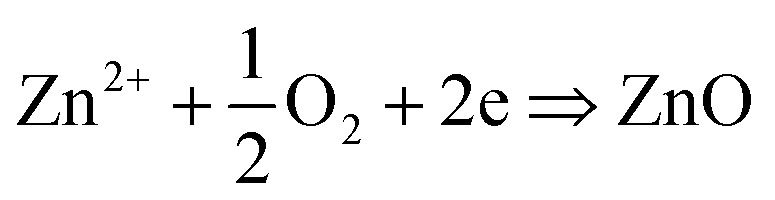


The morphology of the electrosynthesized ZnO thin films was analyzed using a field emission scanning electron microscope (SEM-FEG) ULTRA 55 ZEISS. The crystal structure of the ZnO thin films was investigated by *Empyrean Panalytical* X-ray diffraction (XRD). The wettability of the synthesized thin films of ZnO was quantified by measuring the CAs (drop shape analyzer, Krüss, DSA100). The CA value of each sample represents the average of five measurements at different locations on the surface.

## 3 Results and discussion

The electrodeposited ZnO surfaces with different sizes of nanorods were synthesized from an aqueous electrolyte saturated with O_2_ containing 2.5 mM of ZnCl_2_, 0.1 M of KCl, at *T* = 80 °C for a potential of −1 V/SCE by varying the synthesis time.

### 3.1 Surface characterization

Wettability is a characteristic mainly depending on the surface’s properties that is why we focused on the surface morphology from which the roughness and the fraction of the constitutive elements of the surfaces were estimated.

#### 3.1.1 Surface morphology

**Fig. 1:**
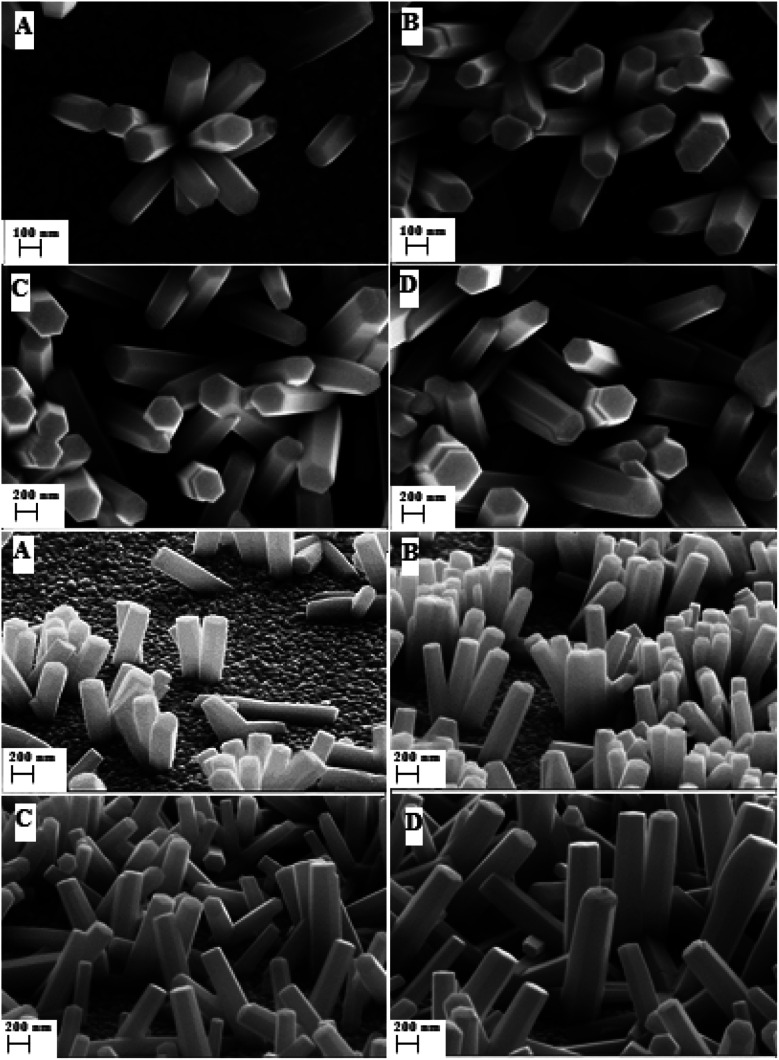
SEM images of electrosynthesized ZnO surfaces (plan and cross-sectional views) of different samples, (A), (B), (C) and (D) corresponding to synthesis times of 1000 s, 3000 s, 5000 s, and 7000 s respectively.

SEM images show the morphologies of the synthesized nanorods of ZnO with different synthesis times (Fig. 1). Two major variations are observed in the ZnO nanorods growth. The first one is related to the nanorods density. From samples A to B, we have an important variation in the density of the ZnO nanorods. However, the density remains almost constant for samples B to D, but a slight broadening of ZnO nanorods is observed. The second major variation concerns the height of the ZnO nanorods. The height of the nanorods increases from 390 nm for sample A to 910 nm for sample D. This time effect on ZnO nanorods growth has been reported previously by Ghannam *et al.*^25^

**Fig. 2:**
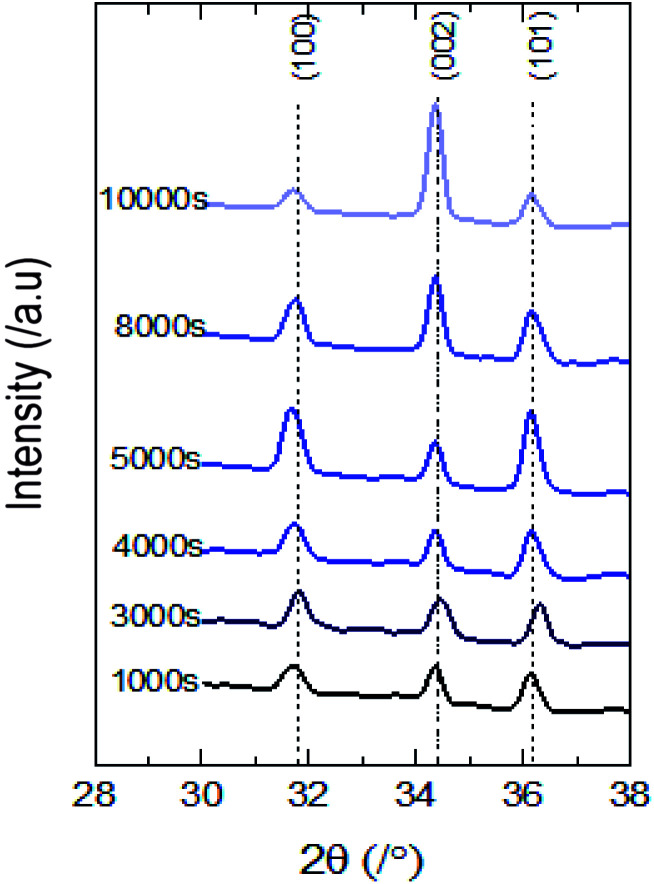
XRD patterns of synthesized ZnO nanostructured thin films.

The distinct X-ray diffraction patterns of all synthesized samples show the appearance of three main intense peaks located at 2*θ* values of 31.8°, 34.4° and 36.3° matching with the hexagonal wurtzite phase of ZnO (Fig. 2). The featured peaks correspond to the (100), (002) and (101) crystal planes respectively.

#### 3.1.2 Surface fraction

The synthesized surfaces are characterized by the surface fraction of the different constitutive elements which are FTO, the polar (P) facet of ZnO, and the non-polar (NP) facet of ZnO.^6^ The surface fraction of a given element x, (*f*_x_), is a dimensionless number equal to the surface of element x, (*S*_x_), over the real surface (*S*_r_) of the sample which is the total surface with the asperities.2
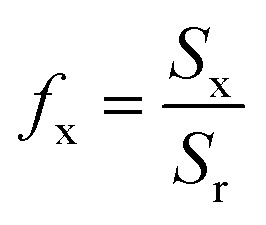


The sum of all elements of the surface fraction is equal to 1.

3 *f*_FTO_ + *f*_NP_ + *f*_P_ = 1

**Fig. 3:**
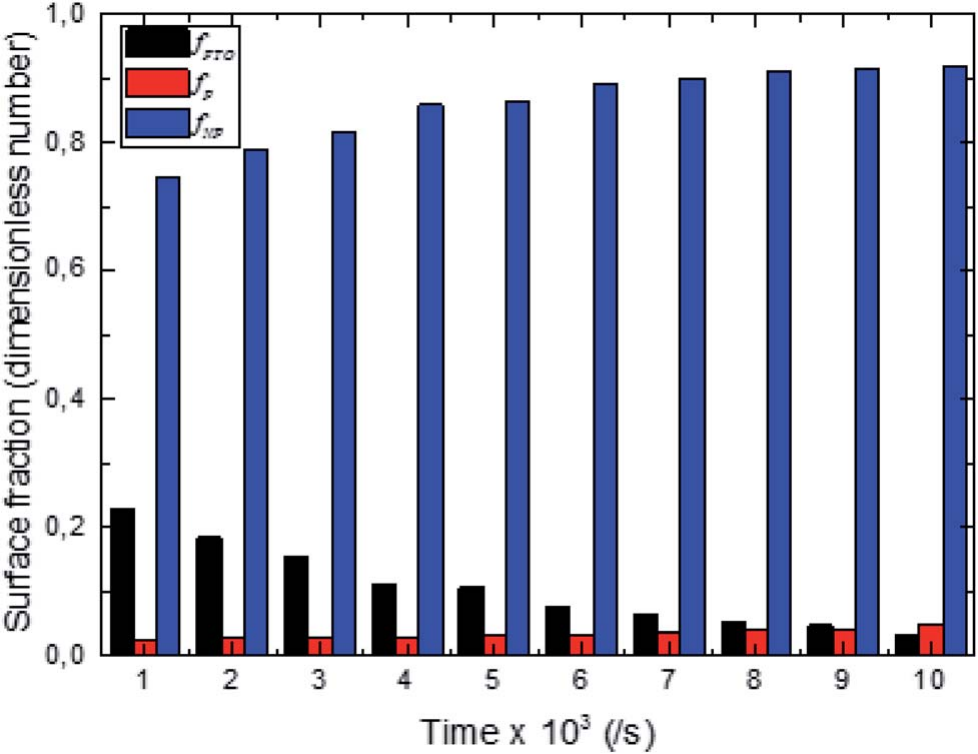
Surface fraction of constitutive elements of the electrosynthesized surfaces (ZnO non-polar facet (*f*_NP_), ZnO polar facet (*f*_P_) and FTO (*f*_FTO_)).

Through the evaluation of the fraction values, we found that the non-polar (NP) surface fraction presents more than 75% of the total surface of the sample. This large value is ascribed to the preferential growth direction of ZnO along the [001] direction. As shown in Fig. 3, the non-polar surface fraction varies increasingly as a function of the synthesis time. It is about 74% for a synthesis time of 1000 s and about 91% for a synthesis time of 10 000 s. However, the polar (P) facet of the ZnO nanorods occupied less than 4.8% of the total surface. It slightly increases as a function of synthesis time and varies from 2.5 to 4.8%. While the FTO surface fraction corresponds to less than 20% of the total surface. It decreases when the synthesis time increases.

#### 3.1.3 Surface roughness

The state of the synthesized surfaces is given through the roughness factor, *r*.^2^ It is a dimensionless number defined as the ratio between the real surface (total area, *S*_R_) and the geometric surface (flat projected area, *S*_A_). The roughness factor takes values superior or equal to 1.4
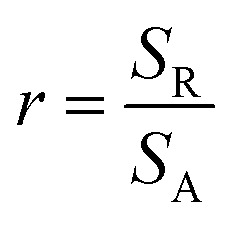


**Fig. 4:**
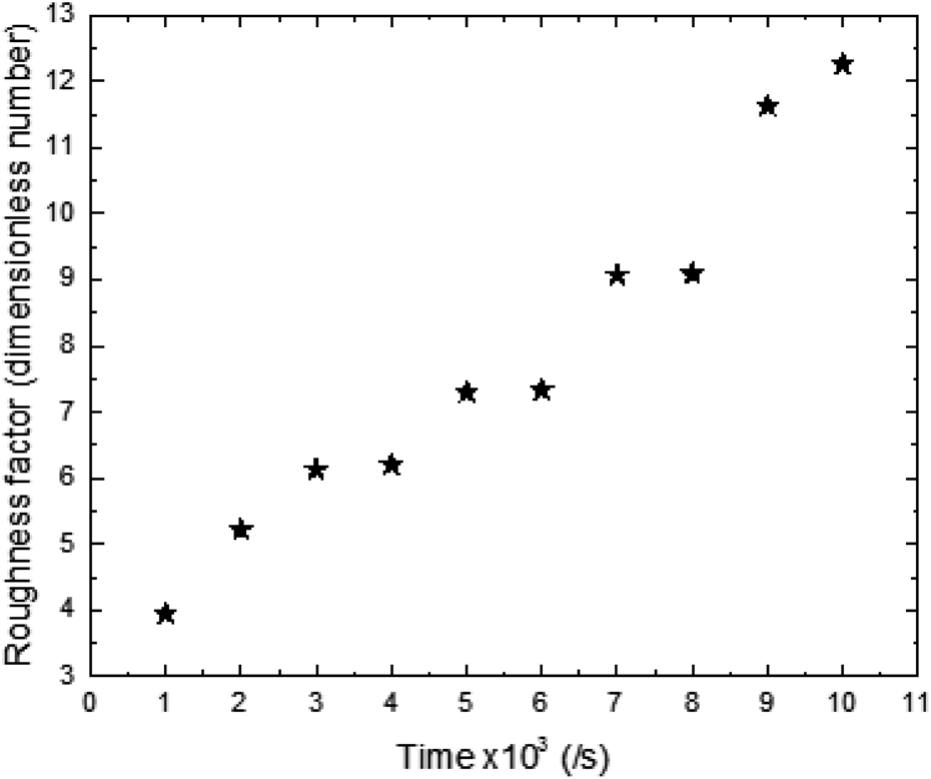
Roughness factor as a function of ZnO electrodeposition time.

The roughness factor of the synthesized surface varies increasingly between 3.94 and 12.26 as a function of time of synthesis (Fig. 4).

### 3.2 Wettability of synthesized surfaces

**Fig. 5:**
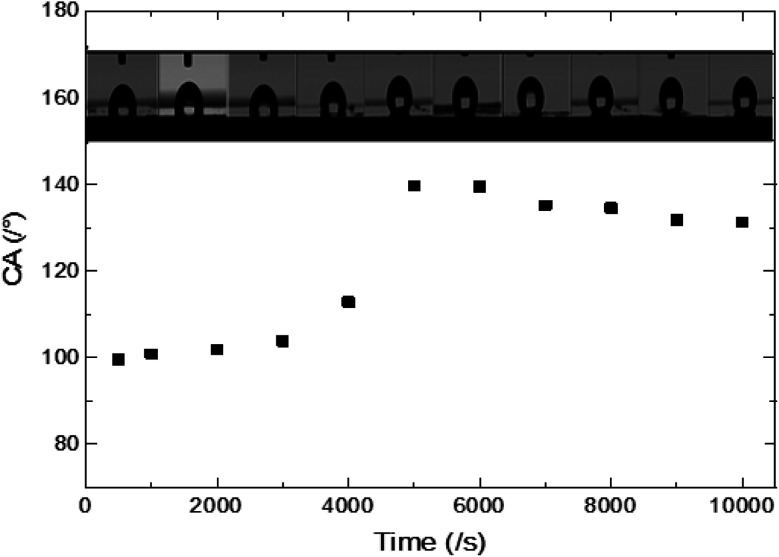
Experimental CAs of liquid drops (3 microliters) on synthesized ZnO surfaces with different synthesis times.

The experimental results of wettability show a small increase in CA for an electrodeposition time between 1000 s and 4000 s. Then we observed a transition, where the CA goes from 117 to 139.6°. This transition is followed by a small decrease in CA for a deposition time higher than 6000 s (Fig. 5).

The surface synthesized at 5000 s exhibits the highest CA (139.6°). Its surface is characterized by a roughness factor of 7.3 and contained 10% of FTO, 2.9% of the polar facet of ZnO and 86% of the non-polar facet of ZnO. While, the surface synthesized at 10 000 s is less hydrophobic despite having a greater roughness factor (about 12.26) and composed of 91.2% of ZnO non-polar facet.

Indeed, the FTO surface is relatively hydrophilic having a CA of 70°. While, the polar surface is normally characterized by a high surface energy thus a low CA is observed. Moreover, the polar facet of ZnO is easily modified by the adsorption of surrounding molecules which contributes to CA modification. The effect of molecules adsorption on the wettability behavior of the ZnO polar facet was observed when we synthesized a smooth layer of ZnO and measured the CA. In the beginning, immediately after the synthesis, the CA of the smooth layer of ZnO is about 0°, but after daily measurements we found that the CA value increases and stabilizes around 90°. However, the non-polar surface has a low surface energy contributing to a high CA value.

Through the CA variation, we deduce that the composition and roughness of the surface modified the state of the liquid drop penetration into the surface asperities contributing to CA modification. In fact, we can point out 3 main states of liquid drop penetration on the ZnO nanorod surface: total penetration, then partial penetration followed by non-penetration (Fig. 7).

**Fig. 6:**
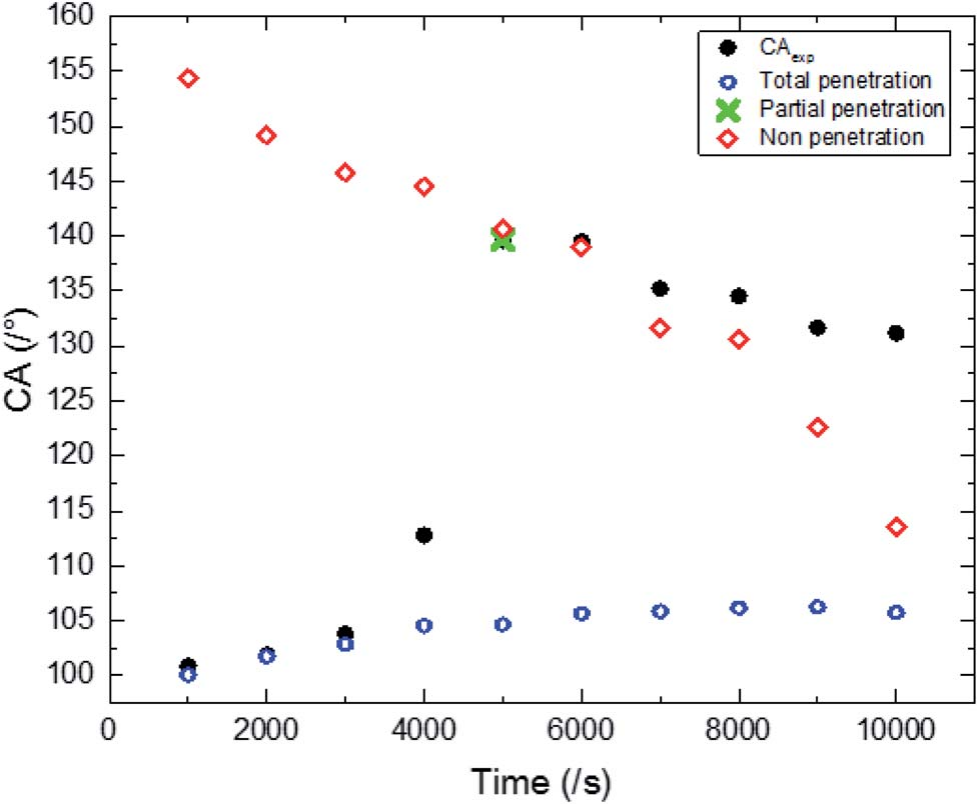
Correlation between experimental CA and theoretical models describing total penetration, partial penetration and non-penetration of liquid drops on ZnO nanostructured thin films with different synthesis times.

**Fig. 7:**
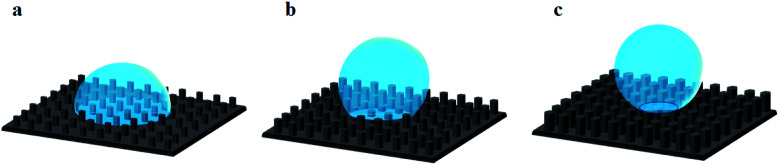
Three main states of liquid drop penetration on a rough ZnO surface. (a) Total penetration, (b) partial penetration, (c) null penetration.

In the first state, total penetration, the liquid drop spreads perfectly in the surface asperities. This state is observed for synthesis times inferior to 4000 s (Fig. 7(a)). Moreover, for this state we observed a strong dependence of CA on surface roughness and its composition (Fig. 6). Where the CA is a result of the combination of Wenzel and Cassie Baxter theoretical models^2,6^ given by the following equation:

5 cos(*θ*_exp_) = *f*_FTO_ cos(*θ*_FTO_) + *f*_NP_ cos(*θ*_NP_) + *f*_P_ cos(*θ*_P_)

The CA of non-polar ZnO is deduced through the experimental CA measurements of total penetration of the liquid drop by applying eqn (5). We found out that the non-polar facet of ZnO is characterized by a CA of 113°.

The second state is the partial penetration (Fig. 7(b)). The abrupt transition of CA value from 112° to 139.6° is due to partial penetration of the liquid drop. The partial penetration arises due to the replacement of the hydrophilic FTO surface by air pockets having a CA of 180°.^6^ This state starts with cancellation of the FTO surface effect and ends with the cancellation of the non-polar facet of ZnO. The partial penetration can be examined by the *x* variation defined as the surface deleted from the NP surface facet thanks to pressurized air. *x* is estimated through experimental CAs by the following equation:6
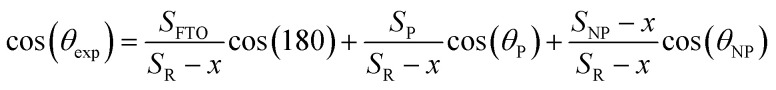


Therefore *x* is given by the following equation:7
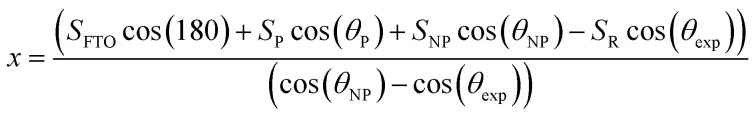


To have partial penetration, *x* must be inferior to the surface of the non polar facet. This condition is theoretically observed only for the sample synthesized at 5000 s.

The third state is non-penetration (Fig. 7(c)). The liquid drop is generally in a Fakir state. The liquid drop is deposed on top of the nanorods. In this case, the surface is composed only by the polar facet of ZnO and gas. The CA for the non-penetration state is given by the following equation:8
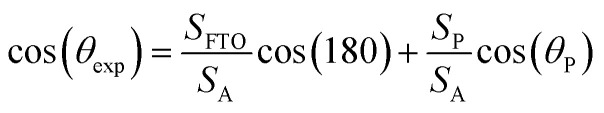


The liquid drop penetration mainly depends on the chemical nature of the element surface composition and the nanorods disposition.^26^ It is important to note that as a function of time ZnO nanorods become very close. The condensation of ZnO nanorods generates a strong repulsion charge for the non-polar facets. The repulsion charge becomes more important when the distance between non-polar facets decreases which contributes to the deformation of the local curvature of the interface separating the liquid/gas resulting from the capillary force at the triple lines. While a water drop can be then pulled from the inside of the cavity if the CA formed between the liquid and solid substrate exceeds 90°.^27^ The deformation of curvature leads to an increase in the liquid/gas interface area underneath the liquid drop contributing to an increase in the surface hydrophobicity. This phenomenon justifies the difference between the experimental CA and that calculated from the non-penetration state for electrodeposition times superior to 7000 s (Fig. 6).

The imperfection of the surface is ascribed to its chemical and physical heterogeneity and can be estimated through the contact angle hysteresis (Δ*θ*) defined as the difference between the advancing (*θ*_a_) and receding (*θ*_r_) contact angles.^28^ The contact angle hysteresis (CAH) can be measured using the tilting cradle method and volume changing method.^29–31^ It has been reported that the tilting cradle method is not suitable for some hydrophobic rough surface due to the friction force that modifies the liquid drop shape.^32,33^

The results for the CAH using the volume increasing/decreasing method show two main behaviours (Table 1). The first one is observed for electrodeposition times varying between 1000 s and 4000 s where the CAH increases from 13 to 28°. The evolution of CAH is mainly due to the surface roughness and the difference in the chemical nature of the constitutive elements of the surface. This heterogeneity contributes to pinning the liquid drop at the surface asperities. In the case of total penetration, the effect of liquid drop pinning on CAH becomes more important when the surface roughness increases. Moreover, physical texturing of the surface seems to have a minor influence on the CAH because the wall of ZnO nanorods responsible for the roughness is non-polar. While the chemical texturing has a major influence on CAH because the drop remains stuck thanks to the polar facet of ZnO and FTO surface. A second evolution is observed in the case of partial/null penetration; for times superior to 5000 s, the CAH decreases taking a value around 7°. The effect of the chemical and physical heterogeneity of the surface which is governing the pinning and sticking phenomena becomes less important and dissipation of the advancing and receding contact angles decreases.

**Table 1:** Advancing, receding and hysteresis contact angles (*θ*_a_, *θ*_r_, Δ*θ*) of ZnO nanorods synthesized with different electrodeposition times

Time × 100 (s)	10	20	30	40	50	60	70	80	90	100
*θ*_a_ (°)	102	105	115	117	142	141	136	135	132	132
*θ*_r_ (°)	89	90	91	89	135	134	129	128	124	124
Δ*θ* (°)	13	15	24	28	7	7	7	7	8	8

**Fig. 8:**
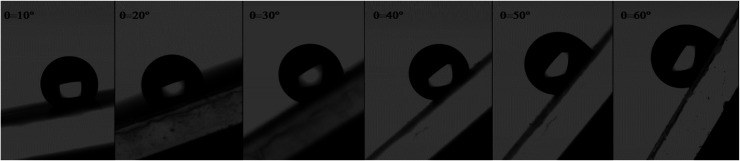
Adhesion of water liquid drop on the ZnO nanorod surface under the gravity effect on an inclined plane.

**Fig. 9:**
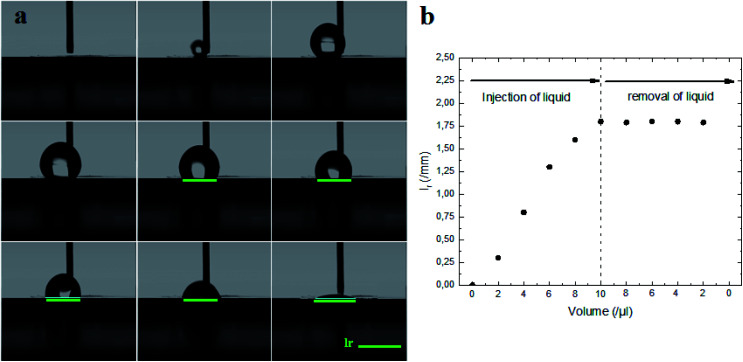
(a) Measure of hysteresis on the ZnO nanorod surface by using the volume increasing/decreasing method. (b) Length of contact line (*l*_r_), formed between liquid and ZnO nanorod surface, measured during injection/removal of liquid as a function of the liquid drop volume.

Indeed, the adhesion of the liquid drop on the super-hydrophobic ZnO nanorod surface, *θ* = 154°, on a substrate inclined at 90° was observed in previous work presented by Kim *et al.*^9^ The adhesion of the liquid drop is also observed in our work on the most hydrophobic surface under gravity of an inclined plane (Fig. 8). The liquid drop on the ZnO nanorod surface remains hanging devoid of any dynamic movement of sliding or rolling. The attachment of the drop on the surface is well observed also by measuring the contact angle advance and retreat by the injection of liquid followed by suction (hysteresis) (Fig. 9(a)). By injecting the liquid to the maximum volume, the length of the contact line (*l*_r_) increases. However at the suction stroke, the line of contact remained the same length. This confirms the hanging of the drop on the surface (Fig. 9(b)).

**Fig. 10:**
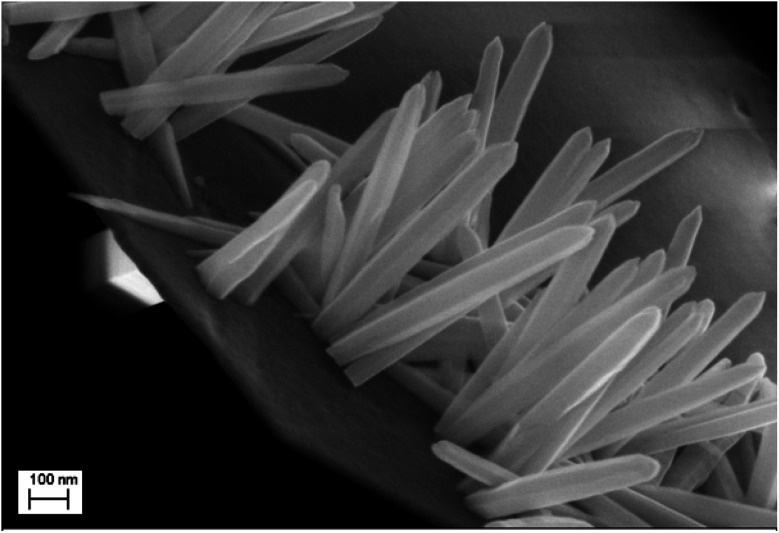
SEM image of ZnO with double scale roughness; microsheets on which nanowires are deposited.

The most demanding condition to have the self-cleaning effect based on super-hydrophobic surface behavior is the liquid drop rolling. Despite the super-hydrophobic comportment of ZnO nanorods, the surfaces lack the self-cleaning property because of the polar facet of ZnO that attracts the water molecules. In order to promote the rolling of the drop on the ZnO surface, the minimization of the polarity of the polar facet of ZnO is necessary. This can be done by chemical modification^34,35^ and/or physical modification of the ZnO surface through the synthesis of ZnO nanopencils, nanotubes or nanowires, as it is also possible by increasing the non-polar ZnO surface, in contact with the liquid, through synthesis of ZnO microsheets on which ZnO nanowires are grown (Fig. 10). Furthermore, the double scale roughness is one of the characteristics observed in Lotus leaf.

**Fig. 11:**
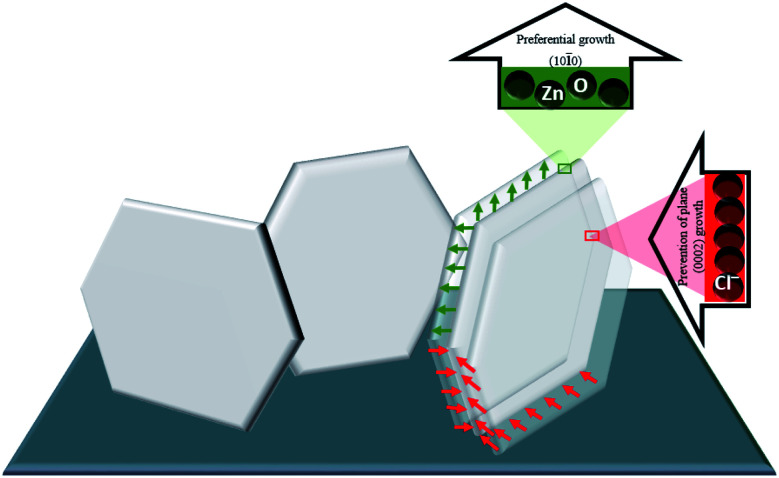
Schematic illustration of ZnO microsheets growth.

According to the literature, the ZnO microsheets are the results of the adsorption of some ions, most likely Cl^−^ ions, on the polar facets of ZnO. The source of Cl^−^ ions can be the Zn^2+^ precursor, ZnCl_2_, also often from KCl or NaCl added to deposition bath to improve the solution conductivity. Most of the solutions used for synthesizing the microsheets of ZnO contain a relatively high concentration of Zn^2+^ precursor (>0.05 M) and a high concentration of Cl^−^ ions (>0.1 M).^22,23,36^ In our case, the microsheets of ZnO were successfully obtained from an aqueous electrolyte saturated by bubbling oxygen, composed of 2.5 mM of ZnCl_2_, 0.5 mM of Al(NO_3_)_3_ and 0.1 M of KCl, with potential and temperature parameters being held constant, −1 V/SCE and 80 °C respectively. For our conditions, the strong adsorption of Cl^−^ provided by KCl and ZnCl_2_ acts as a capping agent which enhanced the growth along the (0002) plane. Whilst the growth along the (101̄0) plane is favored thanks to the high rate of generation of OH^−^ from both molecular oxygen and nitrate, then the Zn^2+^ ions are quickly consumed by OH^−^ ions formed around the (101̄0) plane (Fig. 11). It is important to note that the growth of microsheets through our solution is fast compared to the literature.^22,23,36^

**Fig. 12:**
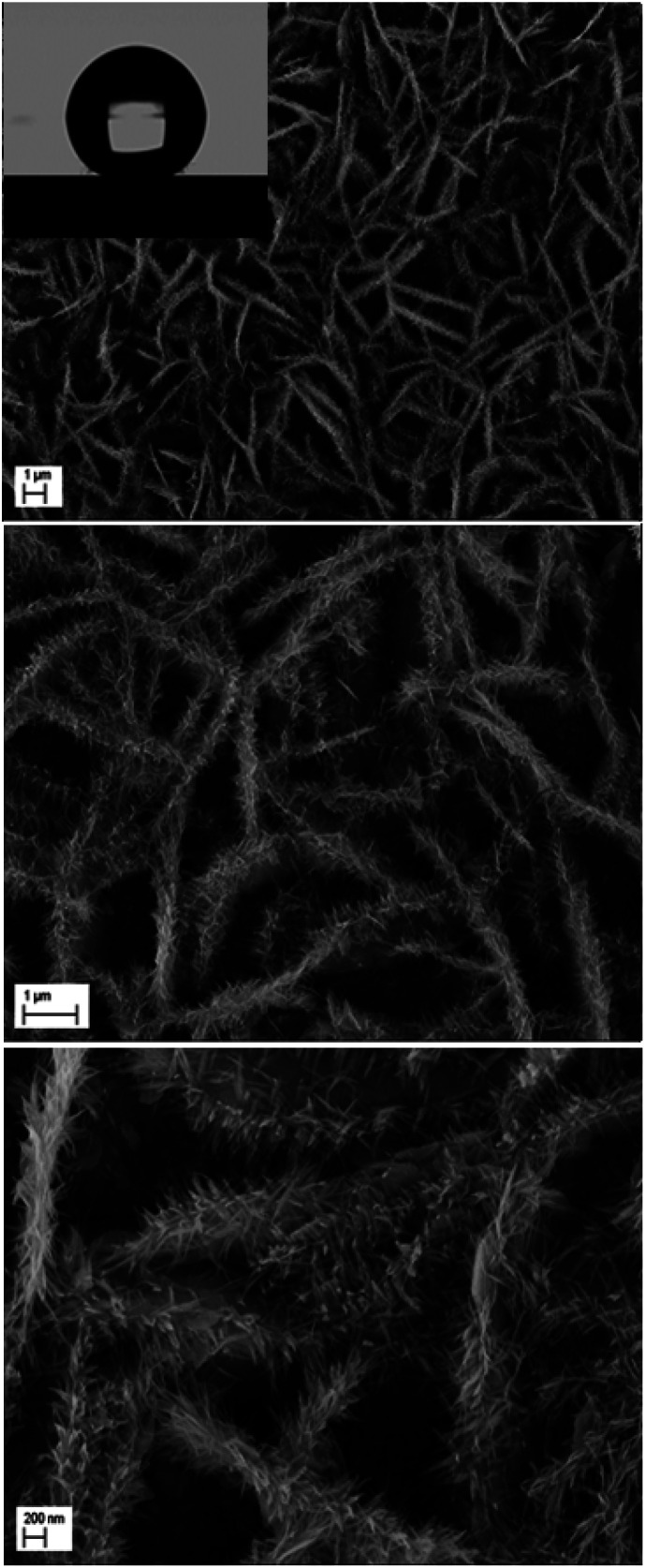
Low and high magnification SEM images of ZnO with double scale roughness: microsheets (*t* = 1000 s) + nanowires (*t* = 5000 s).

The second scale of roughness is the nanowires. Through the studies performed by many groups, the synthesis of ZnO nanowires is carried out using a range of concentrations of Zn^2+^ precursor from 5 × 10^−5^ M to 0.02 M.^37,38^ Elias *et al.*^37^ and Tena-Zaera *et al.*^38^ have discussed the mechanism of growth of ZnO nanowires. Indeed, the diffusion coefficient of Zn^2+^ is much lower than molecular oxygen leading to a fast production of OH^−^ ions. Therefore, the majority of Zn^2+^ ions, which are in low concentrations in the solution, are consumed in a reaction with OH^−^ adsorbed on the tips of nanowires. In our case, the nanowires are obtained from a solution of 0.2 mM ZnCl_2_ and 0.5 M KCl with a potential of −1 V/SCE and a temperature of 80 °C. To ensure the growth of ZnO nanowires on ZnO microsheets and not on the surface of FTO, it is necessary that the synthesis of nanowires be directly after the synthesis of microsheets. The polar facet of ZnO will keep its polarity in order to receive the deposition of ZnO nanowires. The SEM images show the result of the microsheets synthesis followed directly by the synthesis of nanowires (Fig. 12).

**Fig. 13:**
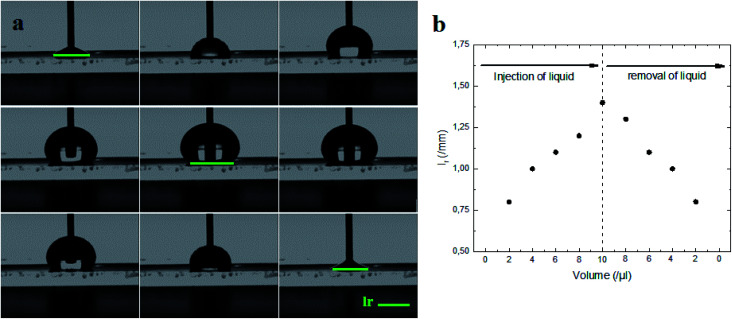
(a) Measure of hysteresis on the superhydrophobic ZnO surface by using the volume increasing/decreasing method. (b) Length of contact line (*l*_r_), formed between the liquid and superhydrophobic ZnO surface, measured during injection/removal of liquid as a function of the liquid drop volume.

**Fig. 14:**
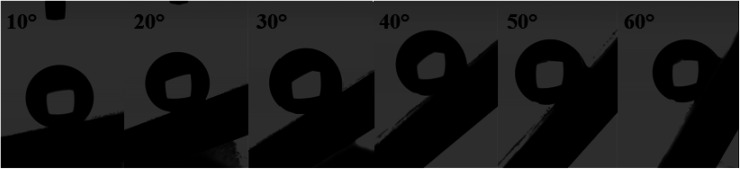
Rolling of the liquid drop on the superhydrophobic ZnO surface.

The surface synthesized by using double scale roughness exhibits a CA of 153° and CAH of 3°. On the other hand, by injecting and aspiring the liquid on the surface, we found that the length of the contact between the liquid and the solid (*l*_r_) varied symmetrically (Fig. 13(a)). During the injection, the length of the contact line increases. However it decreases during aspiration (Fig. 13(b)). This shows the absence of hanging of the liquid drop, hence the rolling of the drop for an inclination angle of the surface superior to 10° (Fig. 14).

## 4 Conclusions

In this work, surfaces of ZnO nanorods with different sizes were synthesized using an electrochemical method. The variation of the physical and chemical texturing of the ZnO surface causes a variation in the CA mainly due to a difference in the liquid drop penetration. By increasing the size of the ZnO nanorods, the surface roughness increases and the fraction of free starting surface, FTO, decreases. While the fractions of the polar and non-polar ZnO facets increase. Indeed, the non-polar facets fraction is more dominant, consequently the surfaces exhibit a hydrophobic behavior. Through the chemical texturing of the surfaces, (*θ*_FTO_ = 70°, *θ*_NP_ = 113°, *θ*_P_ = 90°), partial penetration of the drop was clearly observed. Once the drop partially penetrated a sharp increase in CA was observed from 112° to 139.6°. This increase is related to the substitution of the hydrophilic FTO surface by air pockets. Moreover, two main evolutions of the contact angle hysteresis (CAH) are depicted. For the first evolution, in the case of total penetration of the liquid drop, CAH is mainly governed by the pinning and sticking effect and it is about 13–28°. For the second evolution, the CAH decreases (≈7°) because the pinning effect due to surface roughness is minimized when the liquid drop goes from partial penetration to non-penetration. Moreover, the large variety in CAH is mainly due to physical and chemical surface heterogeneity which contributes to the pinning and sticking of the liquid drop. On the other hand, ZnO with double scale roughness reveals a solution for the rolling of the liquid drop.

## Conflicts of interest

There are no conflicts to declare.

## Supplementary Material
